# Frequency, Co-morbidities and outcome of acute kidney injury in elderly admitted patients

**DOI:** 10.12669/pjms.36.5.2429

**Published:** 2020

**Authors:** Huma Mamun Mahmud, Hormatz Diar Dara Dastoor

**Affiliations:** 1 Dr. Huma Mamun Mahmud, FCPS Nephrology., Department of Medicine, Division Nephrology Al Rahba Hospital, Seha, Abu Dhabi, UAE; 2 Dr. Hormaz Dastoor, MD Nephrology., Department of Medicine, Division Nephrology Al Rahba Hospital, Seha, Abu Dhabi, UAE

**Keywords:** Acute kidney injury, Old age, Outcome, Elderly, Death

## Abstract

**Objectives:**

To analyse the frequency, co morbidities and outcome ofAcute Kidney Injury (AKI) in elderly patients.

**Methods:**

This is an observational retrospective study performed in a secondary care Hospital located in Abu Dhabi. Study include adult patients admitted during 01^st^ January 2018 till 31^st^ December 2018. We uses KDIGO criteria of more than 26.5 micromol/L increase in serum creatinine within 48 hours or 1.5 times increase from baseline within seven days to define AKI. Patients were followed from admission till discharge from hospital either to Home, to other facility or death. Analysis was done on SPSS version 20.

**Results:**

Total admissions for age 60 and above were 880. AKI was observed in 71 patients (8.0%). Most common co-morbidity found was hypertension being observed in 85.9%. Renal recovery was observed in 70.4% out of whom complete recovery was seen in only 32.4% and rest shows partial recovery. Hemodialysis was required in 16.90% and death results in 22.5% of our study patients.

**Conclusion:**

AKI is frequent among elderly admitted patients, commonest co- morbidity in these patients is hypertension. Outcome of AKI in elderly patients is poor with death in 22% of our patients.

## INTRODUCTION

Acute Kidney Injury (AKI) is a rapid decline in renal functions. KDIGO (Kidney disease initiatives in global outcome) guidelines defines AKI as increase in SCr(serum creatinine) by more than or equal to 0.3 mg/dl (or 26.5 micromol/L) within 48 hours or equal to or more than 1.5 times of baseline, known or presumed to have occurred within the prior 7 days.^1^ AKI is reported in approximately 20% of hospitalized patients.^2^

A number of studies define AKI in different clinical conditions. AKI often is result of volume depletion, infections^3^, drugs^4^, contrast induced nephropathy, rhabdomyolysis^5^_,_ obstruction, vasculitis or autoimmune disaeses. A number of clinical chronic condition are also associated with acute and chronic renal failure and has been studied extensively like in heart failure and cirrhosis.^6^ Advancing age is a significant risk factor for renal failure. kidney function declines with increasing age and at age above 40 there is a decline of 10 ml/min/decade.^7^

Declining kidney functions, co-morbidities and poly pharmacy in old age increases the risk of acute injury in elderly patients and even minor insults to aging kidneys results in AKI as function is already compromised. Data is limited for renal failure management in old age, also treatment options are limited as presence of multiple co-morbidities often limit the use of different modes of renal replacement therapies in the elderly patient compromising the renal care.^8^

Incidence of AKI has been defined in one study by Feest TJ, as high as 17 per million yearly in adults under 50 age to 949 per million yearly in 80-89 age group and prostatic disease was found a frequent cause of AkI in old age seen in 25%. Same study also define overall survival was 54% at 3 months.^9^

Another study defines impaired recovery of AKI in aged patients, including data from 17 studies. This study defined that no renal recovery was in 26% in young adults and 31.3% of elderly patients. Limitations of study were variable definitions of AKI, pathogenesis of AKI, criteria of renal recovery, heterogenecity of co-morbidities and use of different study designs.^10^

In our study we attempt to analyse AKI among eldrely patient who required hospital admission. We assessed frequency, co-morbidities, causes, renal survival and the patient survival in these elderly patients as data on AKI is limited in elderly patients.

## METHODS

This is a simple, observational, Single centre study conducted at Al Rahba Hospital, Abu Dhabi with patient induction duration of one year from 01-01-2018 till 31-12-2018. Data collection was done retrospectively. Study started after getting approval from hospital research and ethical committee.

Our study population includes all admissions of adults above 18 years age during specified year for calculation of frequency of AKI in all above 18 age adults. Then we looked at total number of admissions of patients at and above 60 age as elderly age group and we also looked at frequency of AKI in this group to see if the frequency of AKI in all patients and in elderly age group differ. Data for elderly patients was further evaluated in detail for causes and outcome of renal injury in these patients. Data was collected from electronic health record Citrix and was saved electronically. Data was approachable by primary author alone and privacy was maintained. All patient in elderly age group who had AKI admitted and discharged from hospital during specified period were evaluated and included in study population.

AKI was defined as elevation in SCr of equal or more than 26.5 micromol/L within 48 hours or 1.5 times increase in SCr from baseline within seven days as per KDIGO criteria. Demographic data was included and all co-morbidities and cause of renal failure were noted. Recovery was defined as complete if there is complete resolution of AKI and partial if SCr fail to return to baseline SCr. Labs were checked on admission, peak creatinine was marked and SCr was noted at time of discharge or death. We calculated eGFR by (lab information system) through EPI-CKD equation. Record was made of body weight from electronic records. Biochemistry was processed on COBAS 8000 machine. We further scrutinize those patients in elderly age group who had severe renal injury and required hemodialysis during admission and evaluate if they have more risk factors leading to severe injury in them.

Data was analysed on SPSS version 20. All the categorical data was given in percentages and numerical data as mean and standard deviation. Results were given as text, tables and figures.

## RESULTS

Total number of study patient in elderly age group was 71, out of which 41(57.7%) were male. Mean age of patients in elderly age group was 77.20± 10.581, and mean weight was 69.03±22.423. Most of our patients 39 were local belonging to United Arab Emirates (UAE), from Gulf Cooperation Council (GCC) 22 and other expats were 10. Table-I shows that frequency of AKI in our study patients was twice as frequently in elderly age group then in all adults above 18 age.

**Table-I:** Frequency of AKI in all adult admissions above 18 and in elderly age group.

	Total number of hospital admissions	Total number of hospital admissions who had AKI(%)	Number of hospital admissions in elderly age group	Number of hospital admissions in elderly age group who had AKI(%)
Number of hospital admission from 1^st^ January 2018 till 31^st^ December 2018	4424	193(4.36%)	880	71(8.0%)

**Fig.1:**
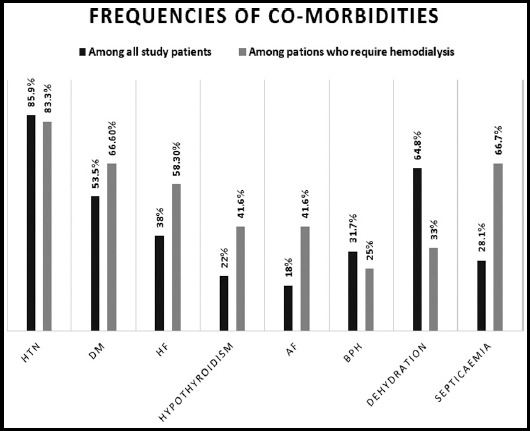
comorbidities among AKI patients.

Numerical data is shown in Table-II. Hypertension was found to be most common associated illness and was seen in 61/71(85.9%), diabetes being second commonest problem seen in 38/71(53.5%). Common co morbidities are given in Fig.1. Most of our patient s were admitted with AKI on admission 52/71(73.2%) and rest of them 19/71 develop AKI during hospitalization.

**Table-II:** Numerical data.

	All AKI study patients (71) Mean±SD	AKI patients who required hemodialysis (12) Mean± SD
Age	77.20±10.581	77.25±9.928
Baseline creatinine	112.50±58.651	148±118
Maximum creatinine	240.42±124.261	375.67±169.947
eGFR before AKI	56.77± 21.706	46.45±24.760
eGFR nadir during AKI	23.89±14.063	7.08±7.179
eGFR on discharge	41.92±27.054	9.5±15.401
Weight	69.03+22.423	66.67±22.89
No of admission days	19.68+ 21.513	44.42+ 28.031

**Fig.2:**
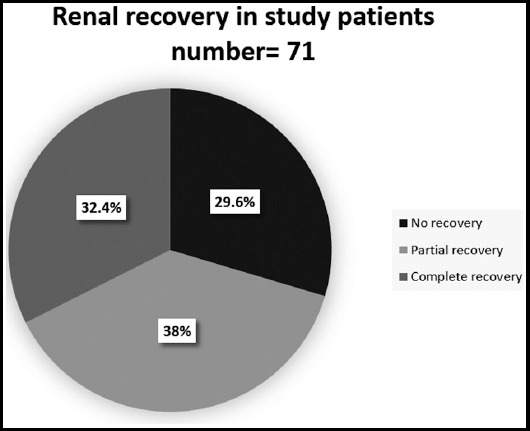
Renal recovery in study patients number=71.

**Fig.3:**
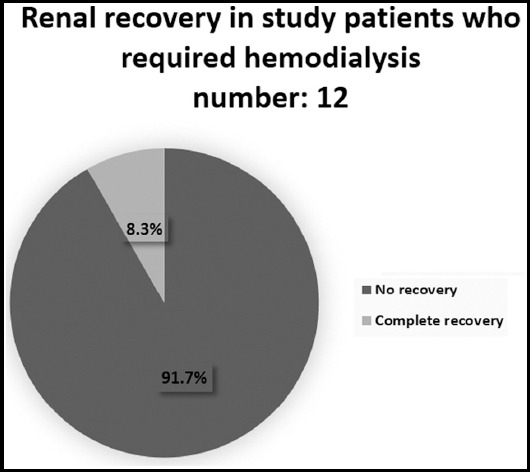
Renal recover in study patients who required hemodialysis number=12.

Among all patient dehydration was seen in 64.8%, 40.8% of patient were on diuretics and 18.3% were on angiotensin converting enzyme inhibitor or angiotensin receptor blocker. Rhabdomyolysis was seen in 2.8%, gastroenteritis in 9.8%, contrast induced nephropathy in 1.4% and no patient had AKI secondary to NSAIDs. We found septicaemia in 28.1% of all patients and 66.7% among those who require hemodialysis. Renal recovery is given Fig.2 and Fig.3. Death was observed in 22.5% of all elderly AKI patients and 75% in those who had severe AKI requiring hemodialysis.

**Table-III:** Complications of AKI.

	All AKI study patients Number=71	AKI patients who required hemodialysis Number =12
Hyperkalemia	15(21.1%)	7(58.3%)
Haemoglobin below 10	26(36.6%)	8(66.6%)
Acidosis	40(56.3%)	12(100%)
Hyponatremia	24(33.8%)	6(50%)
Require mechanical ventilation	23(32.4%)	9(75%)
Death	16(22.5%)	9(75%)

## DISCUSSION

Literature shows patient age above 65 are three times more likely to require hospitalization than those in age 45-64 years, with its attached increased helath cost, also length of stay has been defined to be longer in old age group when compared to young age.^11^

Elderly patients has a number of risk factor which increases their risk of developing AKI, like obesity, atherosclerosis, diabetes, hypertension^12^ and AKI has been defined in elderly population to occur more frequently. Incidence of AKI has been defined in one study by Feest TJ, as high as 17 per million yearly in adults under 50 age to 949 per million yearly in 80-89 age group and prostatic disease was found a frequent cause of AkI in old age seen in 25%. Same study also define overall recovery of 54% at 3 months.^9^

Our study has found frequency of AKI among all adult patients requiring admission in age 18 and above to be 4.36% and frequency of AKI in all those at and above 60 age is 8% which is almost twice the frequency of AKI in all adult admitted patients.

Not too many studies are available to compare frequency of AKI in eldrely patient and most of studies found are prevalence studies. A number of studies has defined that increasing age increases the risk of AKI, and often the presence of multiple co-morbidities and use of multiple drugs in elderly put them at increase risk of AKI^8^, smoking, atherosclerotic disease^13^_,_ Dyslipidemia^15^, Obesity^16^, and male gender^17^ all are associated with an increased risk of renal failure.

Acute kidney injury frequency among hospitalized patient vary from 20-34%.^18^,^2^ In our patient we have found frequency of AKI as 4.36% in all adults which is less than what is defined in other studies. This can be because our hospital is a secondary care hospital with more complicated cases being referred to tertiary care hospitals thereby reducing frequency of acute kidney injury in all hospitalized patients. Also most of the studies giving high frequency of AKI includes patient admission within ICU setting where certainly we see more multiorgan failure patients and higher frequency of AKI whereas our study defines frequency in all admissions to hospital whether in ICU or in general ward.

We found that 73.2% of our study patients with AKI in elederly age group already had AKI on admission. In one of study NSAID induced renal amage was defined to be seen in 15.3% of all cases of drug-induced AKI, and accounted for 25% of cases in those age 65 yr and above.^19^ we do not see NSAIds induced renal injury and attribute it to improving aware ness and cautious use of NSAIDs than previously. About other cause, AKI secondary to contrast was seen in only one patient and rhabdomyolysis was the cause in two of our elderly age group patients.

Septic AKI is independently associated with higher odds of death and longer duration of hospitalization.^3^ In our study we identified sepsis as a major cause of AKI in elderly, frequency being 28.1% and among severe AKI study patients requiring hemodialysis sepsis alone was cause in 66.7% of patients. Also we identified dehydration for any reason was present in 64.8% of our patients with AKI which can also be explained by the fact that 33.8% of these 71 patients were with a past history of CVA and 66.7% of them were already bed ridden when admitted and were dependant on care taker for their food and water intake which make them precarious for dehydration.

We found no patient with AKI secondary to glomerular disease and, post renal causes were also not identified in any of our patients and we may relate it to small number of study population.

Mortality in AKI has been defined variably in different studies varying from 40-60%^18^,^20^,^21^ one of studies shows as high as 75%.^12^ Most of mortality data in studies available is for patient who were critically ill and patients were in ICU settings. AKI has been defined to be an independent mortality risk.^22^,^23^ We have found that among our patient mortality was seen in 22.5% among all elderly patients and it was seen in 75% among those who had severe AKI and who required hemodialysis. This high mortality of 75% among our hemodialsysis requiring patients is same like defined in most of studies involving studies of AKI among critically ill ICU patients.

Among elderly patients who were having less severe degree of renal injury, few of them were dehydrated, were getting diuretics or suffered mild infections and they heal rapidly once offending agent was removed. Among our surviving elderly patients with AKI renal recovery was observed in 72.4% in total out of whom complete recovery was observed in 32.4% only. Among 12/71, who required hemodialysis only 8.3% shows complete renal recovery.

### Limitation of the study

We have not studied causes of AKI in younger patients who are more likely to have these etiologies like trauma and exposure to hard working conditions in extreme of high temperatures in UAE resulting in more frequency of AKI secondary to rhabdomyolysis. Our study population involves elderly who were less exposed to physical stress, trauma, and heat exhaustion.

## CONCLUSION

AKI is twice as common in elderly as in general adult population. Hypertension is the commonest co-morbidity found. Septicaemia and dehydration were seen frequently as a contributory factor in renal failure among elderly. Outcome of AKI in elderly patients is poor with death in 22% of our patients.

### Authors’ Contribution

**HMM:** Conceived, designed, collect data and data analysis, manuscript writing.

**HD:** Contributed to data analysis, review and final approval of manuscript.
